# Primary Gastric Lymphoma: Conservative Treatment Modality Is Not Inferior to Surgery for Early-Stage Disease

**DOI:** 10.5402/2012/951816

**Published:** 2012-08-29

**Authors:** Fatih Selçukbiricik, Deniz Tural, Olgun Elicin, Selin Berk, Mustafa Özgüroğlu, Nuran Bese, Burhan Ferhanoglu

**Affiliations:** ^1^Division of Medical Oncology, Department of Internal Medicine, Cerrahpasa Medical Faculty, Istanbul University, 34098 Istanbul, Turkey; ^2^Department of Radiation Oncology, Cerrahpasa Medical Faculty, Istanbul University, 34098 Istanbul, Turkey; ^3^Division of Medical Oncology, Department of Internal Medicine, Cerrahpasa Medical Faculty, Istanbul University, 34098 Istanbul, Turkey

## Abstract

*Objectives*. The aim of this study was to evaluate clinical characteristics, prognostic factors, survival rates, and treatment modalities in patients with primary gastric lymphoma (PGL). *Methods*. We retrospectively reviewed and analyzed data from patients treated for PGL in our clinic from 1998 through 2010. Staging was performed using the Lugano Staging System. Overall and disease-free survival (OS and DFS) were calculated from the date of diagnosis. *Results*. We identified 79 patients. Thirty-seven patients (47%) were male. The median age at presentation was 57 (18–85) years. The median follow-up time was 41 (9–52) months. Thirty patients (38%) underwent surgery, 74 (92%) received chemotherapy, and 18 (23%) received radiotherapy. The five-year OS and DFS rates were 91.2% and 83.9%, respectively, in patients with stage I/II or IIE disease and 70.6% and 65.5%, respectively, in patients with stage IV disease (*P* = 0.02 for both rates). Treatment modality (surgical or conservative) had no impact on OS or DFS in early stages. In a multivariate analysis, poor performance status, advanced stage, and high LDH levels were significant bad prognostic factors for DFS, while advanced stage, poor performance status, and age > 60 years were significant bad prognostic factors for OS. *Conclusion*. Surgery provides no advantage for survival over conservative treatment; thus, conservative treatment modalities should be preferred initially at early stages of PGL.

## 1. Introduction

Approximately 40% of all non-Hodgkin lymphomas (NHLs) occur in extranodal locations. The gastrointestinal tract is the predominant site of extranodal NHL [1]. Primary NHLs of the gastrointestinal tract are rare, accounting for only 1–4% of malignancies arising in that area [2]. The stomach is the most common site of NHL [3]. In two large studies, primary gastric lymphoma (PGL) accounted for 68–75% of patients with primary gastrointestinal tract NHL [4, 5]. There was a slight predominance of males with PGL (male: and female, 1.1 : 1) [4]. PGL diagnoses reached their peak incidence in individuals between the ages of 50 to 60 years. The most common presenting symptoms included epigastric pain, epigastric discomfort, anorexia, weight loss, nausea, vomiting, and gastrointestinal bleeding. Systemic B symptoms occurred in 12% of PGL patients [4, 5]. 

 Extranodal marginal zone B-cell lymphoma of the mucosa associated lymphoid tissue (MALT) type accounted for 40–48% of PGL cases [4, 5]. According to pathological classification, PGL comprises low-grade and aggressive types. Surgery was the initial treatment for PGL in the past. Currently, high-grade stage I/II PGL can be treated with chemotherapy followed by radiotherapy, and advanced stage PGL is usually treated with chemotherapy only [6, 7]. Surgery is reserved for complicated disease and residual disease after conservative treatment [6]. Low-grade stage I/II *Helicobacter pylori *(+) MALT lymphomas can be treated with antibiotic therapy. Treatment failure with *H. pylori* eradication can be managed with radiation therapy [6].

 In this study, we aimed to determine the clinical characteristics of patients with PGL who were referred to our clinic over the last 12 years. We also aimed to evaluate patient survival and prognostic factors affecting survival and the effect of combined surgical and conservative treatment.

## 2. Materials and Methods

### 2.1. Study Design and Population

We retrospectively reviewed and analyzed the data of patients treated for PGL in our clinic (Istanbul University, Cerrahpasa Medical Faculty, Department of Internal Medicine, Division of Medical Oncology) from 1998 through 2010. All patients with MALT lymphoma who did not respond to antibiotic therapy for *H. pylori* eradication or who demonstrated relapse during followup were included in the study. Approval from the local ethics committee and informed consent of the patients or their next of kin were obtained prior to the study.

### 2.2. Study Procedures

The following characteristics or results were recorded for each patient: medical history, physical examination, biochemistry, computed tomography (CT) of the thorax and abdomen, multiple gastroscopic biopsies of the upper and lower gastrointestinal tract, examination of Waldeyer's ring, and bone marrow biopsy. Disease staging was performed using the Lugano Staging System [7]. Histological diagnosis was determined by skilled hematopathologists. Histological subtype classification was determined according to the World Health Organization criteria [8]. Poor performance status was defined using the Karnofsky scale (<80). A blood lactate dehydrogenase (LDH) level of ≥240 mg/dL was accepted as high (normal range, 0–240 mg/dL). Conservative (e.g., chemotherapy, radiotherapy) or surgical treatments applied to each patient were recorded.

### 2.3. Study Endpoints

Overall and disease-free survival (OS and DFS) were the primary endpoints. OS and DFS were calculated from the date of diagnosis. DFS was defined as the time until disease recurrence, progression, or death from disease or chemotherapy related toxicity, whichever occurred first. OS was defined as the time of death from any cause. The effects of age, sex, LDH level, tumor stage and pathological subtype, performance status, presenting symptoms, and tumor treatment modality on OS and DFS were evaluated. 

### 2.4. Statistical Analysis

Categorical and continuous variables were summarized using descriptive statistics (e.g., median, range, frequency, and percentage) and compared with chi-square and Mann-Whitney *U* tests, respectively. DFS and OS rates were estimated by the Kaplan-Meier method. The effects of clinical variables on DFS and OS were assessed with a univariate analysis. The log-rank test was used to compare curves for the univariate analysis. A Cox proportional hazards model was used to assess independent prognostic factors for DFS and OS. All analyses were performed using SPSS 15.0 (SPSS Inc., Chicago, IL, USA) software. The statistical level of significance was defined as *P* < 0.05. 

## 3. Results

### 3.1. Clinical and Pathological Characteristics

Data from 79 patients with PGL were retrospectively analyzed. The corpus and antrum of the stomach were the predominant sites of PGL. Abdominal pain and dyspepsia were the main symptoms of presentation in 70% of patients, followed by weight loss, fever, or night sweats (B symptoms) in 53% and nausea and vomiting in 10% of patients (Table 1).

 The median age at presentation was 57 years (range, 18−85 years) and 37 patients (47%) were male. Of the 79 patients, 63 (79%) were classified as having non-MALT and 16 (21%) as having MALT lymphoma. Eleven patients had a poor performance status, according to the Karnofsky scale (<80). Of the 79 total patients, 57 (81%) had stage I/II or IIE disease and 22 (19%) had stage IV disease. The clinical characteristics of the patients with respect to MALT status are shown in Table 2. 

### 3.2. Treatment Modalities and Response

The treatment modalities applied to patients with respect to MALT status and stage of disease are shown in Table 3. Thirty patients (38%) underwent surgery, 74 (92%) received chemotherapy, and 18 (23%) received radiotherapy. The most common type of surgical treatment procedure was total gastrectomy and lymph node dissection either as combined or single treatment modalities. The most common chemotherapy regimen was cyclophoshamide, doxorubicine, vincristine, and prednisolone (CHOP) (70% of patients). Fifty percent of patients were administered six cycles of chemotherapy, 25% were administered three cycles, and 25% were administered four or five cycles. Patients who received radiotherapy were treated with extended-field irradiation to the upper and middle parts of the abdomen with 30–45 Gy.

### 3.3. Survival and Univariate Analysis

**Figure 1:**
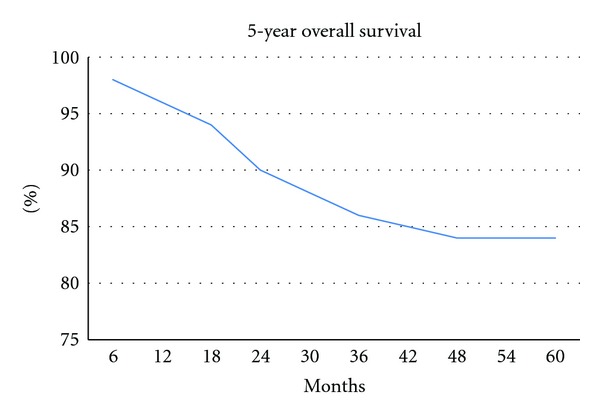
Kaplan-Meier curve for overall survival of 79 patients with PGL.

**Figure 2:**
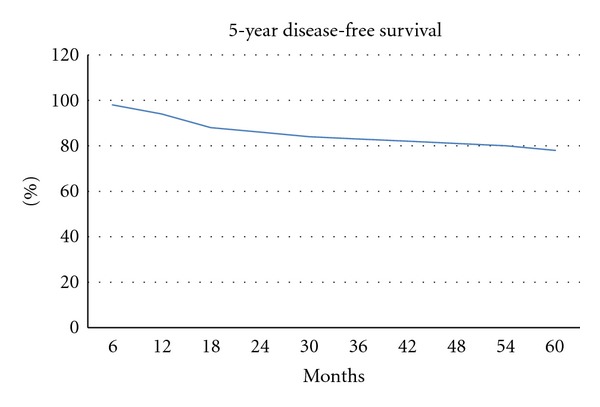
Kaplan-Meier curve for disease-free survival of 79 patients with PGL.

All 79 patients were enrolled into our survival analysis study. The median followup was 41 months (range, 9−52 months). The five-year OS and DFS, which were estimated by using the Kaplan-Meier method, were 83.1% and 78.6%, respectively (Figures 1 and 2). In patients with stage I/II or IIE presentation, the five-year OS and DFS rates were 91.2% and 83.9%, respectively. The five-year OS and DFS in patients with stage IV disease were 70.6% and 65.5%, respectively. Both OS and DFS were significantly lower in those with advanced stage disease (*P* = 0.002 and *P* = 0.02, resp.). For MALT lymphoma patients, the five-year OS and DFS rates were 91.2% and 81.3%, respectively. OS and DFS were not different between MALT and non-MALT groups (*P* = 0.38 and *P* = 0.57, resp.). Poor performance status was significantly associated with lower DFS and OS rates (*P* = 0.002 for both). Patients with high LDH levels had significantly lower DFS (*P* = 0.007). Treatment modality (surgical or conservative) had no impact on OS or DFS. The effects of clinical variables on five-year OS and DFS are given in Table 4. 

### 3.4. Multivariate Analysis for DFS and OS

Based on the results from the univariate analyses, we performed multivariate analyses using a Cox proportional hazard model (Table 5). Poor Karnofsky performance status, advanced stage, and high level of LDH were significant prognostic factors for DFS in the Cox model. Advanced stage, poor Karnofsky index, and age >60 years were significant prognostic factors for OS in multivariate analyses.

## 4. Discussion

PGLs represent more than half of all primary gastrointestinal lymphomas, accounting for 5% of all malignant tumors of the stomach [9–12]. According to histological type, PGLs are divided into low-grade and high-grade. In this retrospective study, the rate of MALT lymphomas (21%) was lower than previously shown [4, 5]. About 40% of PGLs are low-grade lesions thought to arise in the mucosa from the defined MALT lymphoma, and 60% are histologically considered high-grade [4, 6, 10]. 

Epigastric pain, epigastric discomfort, anorexia, weight loss, nausea, vomiting, and gastrointestinal bleeding were the most common symptoms in the present study, as reported in other series [4, 13–15]. In the current report, there was a slight male predominance [4, 13, 14, 16], while other studies reported female prominence among cases of PGL [14, 16].

In PGL, there are multiple factors that affect survival. In previous studies, advanced stage, poor performance status, age >60 years, and elevated LDH at presentation of the disease were associated with poor outcome; female sex, low-grade histology, good performance status, and surgical resection for local disease have been reported to be associated with high OS and DFS rates [4, 17–19]. In our study, the five-year OS and DFS rates were 83.1% and 78.6%, respectively. In patients with stage I/II or IIE disease at presentation, the five-year OS and DFS rates were 91.2% and 83.9%, respectively. The five-year OS and DFS rates of those with stage IV disease at presentation were 70.6% and 65.5%, respectively. In prior studies, the survival rate of patients with PGL was 78–92%, according to stage and treatment modality [6, 19–22]. Based on our results, variables associated with poor DFS were advance stage, high LDH level, and poor performance status. In a multivariate analysis, variables associated with poor DFS were also advanced stage, high LDH level, and poor performance status. Regarding OS, a univariate analysis revealed that advanced stage, age >60 years, and poor performance status were associated with a poor prognosis. In the multivariate analysis, advanced stage, poor performance status, and age were associated with a poor prognosis.

There are no standard therapeutic guidelines for patients in whom antibiotic therapy has failed. In two retrospective studies of patients with MALT lymphoma, no significant differences in survival were found between different treatment modalities [23, 24]. In our study, patients with MALT lymphoma were those in whom antibiotic therapy failed. No significant differences in DFS or OS were demonstrated among PGL patients who had nonsurgical or surgical treatment of the local disease (stage I/II or IIE) of MALT or non-MALT lymphoma. Controversy remains over the optimal treatment for early stages of PGL, particularly regarding the role of surgery. Historically, surgery has been used as the initial treatment for PGL [13]. Recently, it has become evident that there is no difference in survival rates for those who were treated with surgery compared to a conservative modality. Surgery is no longer accepted as the cornerstone treatment of PGL and is reserved for when nonsurgical treatment is not possible. Surgery should be considered following nonsurgical treatment and when disease complications, such as hemorrhage, obstruction, or perforation, have occurred [9]. However, it is well known that surgical approaches have a number of potential disadvantages. Mortality associated with surgery has been estimated at nearly 8% [25]. Additionally, significant morbidity is associated with gastrectomy, as follows: 17% of patients developed malabsorption syndromes, 38% reported weight loss, and 13% developed dumping syndrome [26]. On the other hand, of the patients who received chemotherapy, only 5% developed acute complications, such as gastric perforation and gastrointestinal hemorrhage [20, 27–30].

In the present study, there was no difference between conservative and surgical treatment approaches with regard to DFS and OS. The main limitations of this study were its retrospective design and the lack of data on patient quality of life and toxicities in patients treated with a conservative approach or surgery.

In conclusion, poor performance status, advanced stage of disease, high LDH level, and advanced age were bad prognostic factors for patients with PGL. Surgery provides no advantage for survival over conservative treatment; thus, conservative treatment modalities should be preferred initially at early stages of PGL. Further prospective, large-scale, controlled studies are needed to determine the impact of different treatment modalities on the outcome of patients with PGL.

## Figures and Tables

**Table 1:** Predominant site of primary gastric lymphoma and symptoms at diagnosis (*n* = 79).

	*n*	%
Predominant site of gastric lymphoma		
Corpus	28	35
Antrum	26	33
Cardia	7	9
Fundus	2	3
Multiple site	5	6
Unknown	11	14
Symptoms		
Abdominal pain and dyspepsia	55	70
Weight loss, fever, night sweat	42	53
Nausea and vomiting	13	17
Weakness	11	14
Gastrointestinal bleeding	8	10

**Table 2:** Clinical characteristics of study patients (*n* = 79).

		Non-MALT (*n* = 63)	MALT (*n* = 16)
Age (years, median (range))		53 (43–63)	56 (32–80)
Gender (*n* (%))	Male	28 (45%)	9 (56%)
	Female	34 (55%)	7 (44%)
Stage (*n* (%))	I/II, IIE	44 (71%)	13 (81%)
	IV	19 (29%)	3 (19%)
Karnofsky performance status (*n* (%))	≥80	56 (89%)	12 (75%)
	<80	7 (11%)	4 (25%)
LDH level (*n* (%))	>240 mg	26 (42%)	9 (56%)
	Normal range	28 (45%)	5 (31%)
	Unknown	9 (13%)	2 (13%)

MALT: mucosa associated lymphoid tissue, LDH: lactate dehydrogenase.

**Table 3:** Treatment modality applied for study patients (*n* = 79).

	Non-MALT (*n* = 63)		MALT (*n* = 16)	
	Stage I/II, IIE	Stage IV	Stage I/II, IIE	Stage IV
Conservative	27 (63%)	12 (67%)	7 (54%)	3 (100%)
CT	22	10	3	3
CT + RT	5	2	1	
RT			3	

Nonconservative	17 (37%)	7 (33%)	6 (46%)	0
Surgery + CT	16	6	4	
Surgery + CT + RT	1	1	2	

MALT: mucosa associated lymphoid tissue, CT: chemotherapy, and RT: radiotherapy.

**Table 4:** The clinical variables and their prognostic impact on five-year DFS and OS.

		DFS (%)	*P* value^a^	OS (%)	*P* value^a^
Gender	Female	76.4	0.7	84.1	0.6
	Male	81.2		81.9	
Age	>60	77.9	0.4	75.5	0.04
	≤60	79.9		89.8	
LDH	>240 mg	72.5	0.007	79.9	0.09
	Normal range	86.8		90.9	
Symptoms	B symptoms present	82.3	0.4	79.2	0.2
	B symptoms absent	74		88.6	
Lymphoma subtype	MALT	81.3	0.57	91.2	0.38
	Non-MALT	76.9		81.2	
Karnofsky index	≥80	89.5	0.002	89.5	0.002
	<80	54.5		63.6	
Stage	I/II, IIE	83.9	0.02	91.2	0.002
	IV	65.5		70.6	
Treatment (for stage I/II, IIE)	Surgery	85.7	0.18	86.5	0.8
	Conservative	82.1		94	

^a^Long-rank test.

DFS: disease-free survival, OS: overall survival, LDH: lactate dehydrogenase, and MALT: mucosa associated lymphoid tissue.

**Table 5:** Multivariate analyses for DFS and OS.

		DFS			OS		
		RR	95% CI	*P* value	RR	95% CI	*P* value
Karnofsky index	≥80	1	1.5–10.9	0.005	1	1.6–11.2	0.001
	<80	4.1			4.1		
Stage	I/II, IIE	1	1.2–1.9	0.01	1	1.5–9.7	0.004
	IV	1.4			3.8		
LDH	Normal range	1	1.05–1.63	0.02			
	>240 mg	1.18					
Age	≤60 years				1	1.15–1.9	0.04
	>60 years				1.4		

RR: relative risk; CI: confidence interval, DFS: disease-free survival, OS: overall survival, and LDH: lactate dehydrogenase.
